# Supplementary material for: Dispersal history and bidirectional human-fish host switching of invasive, hypervirulent Streptococcus agalactiae sequence type 283
Source: PLOS Glob Public Health. 2023 Oct 19;3(10):e0002454. doi: 10.1371/journal.pgph.0002454 (PMC10586614; doi:10.1371/journal.pgph.0002454)
Supplement: S1 Text — (PDF) [file pgph.0002454.s001.pdf]

Supporting information for

Dispersal history and bidirectional human-fish host switching of invasive, hypervirulent *Streptococcus agalactiae* sequence type 283

Daniel Schar[a,*], Zhenyu Zhang[b], Joao Pires[c], Bram Vrancken[a,d], Marc A. Suchard[e,b], Philippe Lemey[d], Margaret Ip[f], Marius Gilbert[a,g], Thomas Van Boeckel[c,h], Simon Dellicour[a,d]

[a] Spatial Epidemiology Laboratory, Université Libre de Bruxelles, B1050 Brussels, Belgium;
[b] Department of Biostatistics, Fielding School of Public Health, University of California Los Angeles, Los Angeles, CA 90095, USA;
[c] Institute for Environmental Decisions, ETH Zurich, Zurich 8006, Switzerland;
[d] Department of Microbiology, Immunology and Transplantation, Rega Institute for Medical Research, KU Leuven, 3000 Leuven, Belgium;
[e] Department of Human Genetics, David Geffen School of Medicine at UCLA, University of California, Los Angeles, Los Angeles, CA, 90095, USA;
[f] Department of Microbiology, Faculty of Medicine, Prince of Wales Hospital, The Chinese University of Hong Kong, Hong Kong SAR, China;
[g] Fonds National de la Recherche Scientifique, B1000 Brussels, Belgium;
[h] Center for Diseases Dynamics, Economics, and Policy, New Delhi, India

[*]To whom correspondence may be addressed.
Daniel Schar

Unit 7201 Box 692
DPO AE 09974-0692

Email: dlschar@gmail.com; Daniel.Schar@ulb.be

This file includes:

Methods in S1 Text

Tables A and B in S1 Text

Fig A - E in S1 Text

References in S1 Text

Methods in S1 Text

Whole genome sequence analysis.

A total of 317 sequences were obtained as assemblies, and 11 as whole-genome sequencing reads. Quality of whole-genome sequence data was assessed using FastQC v.0.11.9 [1]. Whole-genome sequence paired-end reads were assembled using SPAdes v.3.15.2 [2] with the "--careful" option. Assemblies were mapped to reference genome SG-M1 (accession CP012419.2) [3] and aligned using SKA v.1.0 [4]. Putative regions of recombination were identified on the resulting 2.12 Mb whole genome alignment using Gubbins [5]. Putative mobile genetic elements (MGE) were predicted and masked from the alignment, after which regions of recombination identified using Gubbins were purged, yielding a MGE-free and putatively non-recombinant alignment. Single nucleotide polymorphisms (SNP) were called on this alignment using SNP-sites [6].

Prediction of mobile genetic elements.

The following mobile genetic elements were predicted in the SG-M1 genome: phages/prophages, CRISPR arrays, and insertion sequences/transposons (Table A in S1 Text).

Phage/prophages were predicted in the SG-M1 reference genome (accession CP012419.2) using the web version of PHASTER (https://phaster.ca/) and were used as reported. CRISPR arrays were predicted using CRT v1.2 [7], CRISPRDetect v2.4 [8], and PILER-CR v1.06 [9]. In all cases, the SG-M1 genome sequence was used as input with default parameters selected. The three sets of results were manually inspected and found to differ by only one divergent repeat sequence in each of the two predicted arrays. We included the coordinates for the divergent

repeat in our final definition of the extent of both arrays. Insertion sequences/transposons were predicted using the ISEFinder database (with the BLASTN tool at https://isfinder.biotoul.fr/blast.php on March 20, 2022) and digIS (cloned from GitHub on March 20, 2022) [10]. The results were manually reconciled yielding a total of 16 predictions, of which 10 predictions were nearly identical using both methods (start and end within 4 bp). For three predictions, one of the programs predicted a sequence that was completely contained in a prediction from the other program; we took the larger prediction in these cases. Two predictions were unique to digIS, and both were ORF predictions (as opposed to complete IS predictions); we accepted these to prevent potentially spurious SNPs from other IS/Tn-related genes that might align to these individual ORFs. Finally, one prediction consisted of an IS predicted identically by both programs, but digIS further predicted a partial ORF that extended beyond one border of the identically predicted IS; we took the union of these coordinates as the final prediction to guard against potentially spurious SNPs.

Prediction of antimicrobial resistance and virulence factor genes.

Sequences were screened for antimicrobial resistance and virulence factor genes with Abricate [11] using the NCBI, Resfinder, ARG-ANNOT and VFDB databases (with database updates available as of 29 March 2022). AMRFinderPlus [12] was used to further screen sequences for both acquired antimicrobial resistance genes and point mutations using the *Streptococcus agalactiae* tag. Results were manually reconciled and genes identified in any of the databases with a minimum coverage match of 80% to the query sequence length were classified as "present"; gene coverage match below this threshold was classified as "absent."

Phylogenetic analysis.

A maximum likelihood (ML) phylogenetic tree was inferred from the 1,214 SNP alignment using RAxML v8.2.12 [13] with a general time reversible (GTR) and gamma model of rate heterogeneity. We performed 100 bootstrap replications to assess internal node support. A root-to-tip regression was performed based on the ML tree to evaluate the temporal signal inherent to our dataset. For this purpose, we used the R package "BactDating" [14] and observed a relatively weak yet supported correlation between date of sampling and genetic divergence ($R^2 = 0.22$; $p < 0.0001$; Fig A in S1 Text). Trees were visualized and annotated using the R package "ggtree" v3.2.1 [15] and the program FigTree v1.4.4 (http://tree.bio.ed.ac.uk/software/figtree/).

Spatio-temporal and discrete trait analyses.

To circumvent convergence issues with a joint inference approach and reduce computational burden, we used a multi-step process to generate spatial reconstructions of GBS ST283 transitions and to infer the gain and loss of antimicrobial resistance and virulence factor genes. In the first step we inferred a time-scaled phylogenetic tree from the ML tree using Markov chain Monte Carlo (MCMC) simulations over 10 million generations in the R package "BactDating," with convergence confirmed through visual inspection of traces and effective sample sizes (ESS) ranging between 204 and 501. In the second step, we used this time-scaled phylogenetic tree as a fixed tree topology to perform both a discrete phylogeographic reconstruction and to analyze gene transition rates using the discrete diffusion model [16] implemented in the software package BEAST v1.10 [17].

Specifically, a Bayesian stochastic search variable selection (BSSVS) approach was used to identify state transitions for discrete traits. Ancestral trait reconstructions were performed for location, antimicrobial resistance genes, and virulence factor genes. The MCMC algorithm was run for two billion iterations, sampling every 10^5 iterations. MCMC convergence and mixing, as well as ESS values associated with estimated parameters were checked using Tracer v1.7 [18]. All ESS values were >200. The maximum clade credibility (MCC) tree was identified and annotated with the program TreeAnnotator v1.10 [17] among 1,000 trees sampled from the post-burn-in posterior distribution.

In the discrete phylogeographic reconstruction, country transitions were estimated as Markov jump counts in the BSSVS analysis. Markov jumps were reported along with both standard Bayes factor (BF) support values (ratio of posterior over prior odds and interpreted as a measure of the strength of evidence for the alternate hypothesis) and an adjusted Bayes factor (BF_{adj}) [19]. As disparity in sample sizes could influence transitions between states, the BF_{adj} value accounts for relative abundance in sample sizes for discrete traits by generating a prior with randomized traits over tree tips (tip swapping) in each MCMC iteration in a manner that approximates the tip-date randomization test for temporal signal [20]. Standard BF and BF_{adj} values >20 were considered strong statistical support [21].

To account for heterogeneous sampling orientation amongst host type in the dataset, we inferred host switching by working on downsampled time-scaled phylogenetic trees obtained by randomly sampling equal numbers of isolates originating from human and fish hosts in the tree tips. We generated 1,000 downsampled phylogenies, performing in each tree a maximum

likelihood ancestral host estimation using an equal rates model implemented in the R package "ape" and counting host state transitions (human-to-fish and fish-to-human). The transition counts in each tree were taken as the distribution from which the median and interquartile range (IQR) were calculated.

As the dataset was over-represented by human origin isolates in the earliest years, we conducted three additional analyses to assess the impact of sampling date on host switching (Fig 3). In the first analysis, we worked with a downsampled dataset consisting of equal numbers of isolates originating from human and fish hosts using only isolates within a window of years where both human and fish host isolates were represented. In the second analysis, we worked with the downsampled dataset from the first analysis, but further time-matched isolates by year and host. Specifically, an equal number of isolates originating from human and fish hosts per year were retained, where that number was defined as the minimum number of isolates available for either one of the two host types in that year. Finally, the third analysis followed the procedure for the second analysis but was based on an original tree that was first subsampled by phylogenetic clustering to account for the uneven phylogenetic diversity associated with each host type – namely, sequences collected in fish having here a greater tendency to cluster within monophyletic clades. Specifically, subsampling by phylogenetic clustering consisted of removing sequences such that monophyletic clusters of sequences collected from the same host type are only represented by a single sequence [22]. In the context of the ancestral reconstruction of the host trait, such clusters would largely represent dispersal within the same type of host. Therefore, performing this preliminary subsampling step prior to the subsampling by year and by host prevents giving too much weight to monophyletic clades gathering several sequences

sampled from the same year and collected within the same type of host. All resulting downsampled datasets were then subjected to ancestral host reconstructions across 1,000 replicates using the method described above.

Across-trait host-gene and gene-gene correlations.

We apply a recently developed phylogenetic multivariate probit model [23,24], which can efficiently learn the correlation between discrete traits while adjusting for across-taxa covariation inherent to the phylogenetic tree. This model assumes continuous latent variables underlying discrete traits where the latent variables follow a Brownian diffusion along the tree. The across-trait partial correlations describe conditional dependencies between any two traits without confounding from other considered traits [23]. As compared with empirical correlations (Fig E in S1 Text), which do not account for the inherent covariation amongst taxa in the phylogenetic tree, these partial correlations provide enhanced insights into potential molecular mechanisms without the confounding influence of the shared evolutionary history between lineages. We performed Bayesian inference of the phylogenetic probit model [23] implemented in BEAST v1.10 [17] and reported the pairwise partial correlations with a posterior median > 0.2 or < -0.2 (Fig 5).

Data analysis was conducted in R version 4.1.3.

Reference	Start coordinate	End coordinate	Mobile genetic element
NZ_CP012419.2	143798	145336	ISSag9 -- IS30
NZ_CP012419.2	165152	166315	ISSag3 -- IS30
NZ_CP012419.2	168722	169589	IS1381A -- IS5/ISL2
NZ_CP012419.2	487105	487966	IS1381A -- IS5/ISL2
NZ_CP012419.2	611044	611990	IS3 orf -- IS3/IS150
NZ_CP012419.2	618464	619236	Partial ISSag5 -- IS3/IS150
NZ_CP012419.2	638397	639656	ISSag5 -- IS3/IS150
NZ_CP012419.2	960119	960980	IS1381A -- IS5/ISL2
NZ_CP012419.2	1113424	1114287	IS1381A -- IS5/ISL2
NZ_CP012419.2	1145429	1146293	IS1381A -- IS5/ISL2
NZ_CP012419.2	1258749	1259968	ISSag2 -- IS3/IS150
NZ_CP012419.2	1259969	1261594	ISSag5/ISStin5 -- IS3/IS150
NZ_CP012419.2	1269478	1270578	ISLre2 orf --
NZ_CP012419.2	1270577	1271707	ISSag4 -- IS3/IS150
NZ_CP012419.2	1952653	1953517	IS1381A -- IS5/ISL2
NZ_CP012419.2	2021576	2022442	IS1381A -- IS5/ISL2
NZ_CP012419.2	81637	124321	PHAGE_Strept_315.5_NC_004588(23)
NZ_CP012419.2	1184424	1221962	PHAGE_Strept_9871_NC_031069(20)
NZ_CP012419.2	1247050	1275385	PHAGE_Bacter_Diva_NC_028788(3)
NZ_CP012419.2	2041942	2058407	PHAGE_Lactoc_bIL310_NC_002669(3)
NZ_CP012419.2	513653	514345	CRISPR Array 1
NZ_CP012419.2	915875	916570	CRISPR Array 2

Table A. Mobile genetic elements (CRISPR, phage, IS/Transposons) predicted in SG-M1. Predicted mobile genetic elements were masked from the whole genome alignment.

	Root node location (posterior probability)	Root node location Tip swapping (posterior probability)
China	0.000	0.002
Hong Kong	0.001	0.004
Laos	0.004	0.052
Malaysia	0.000	0.037
Myanmar	0.000	0.002
Singapore	0.911	0.800
Thailand	0.083	0.102
Vietnam	0.001	0.001

Table B. Phylogeographic analysis of location at the ancestral root node. Posterior probabilities for ancestral root node location are displayed for each country using both standard and tip swapping analyses (see Methods for further detail). Comparatively high posterior probability assigned to Singapore at the root node of the phylogeny using both standard and tip swapping analyses indicates that higher sampling effort in Singapore is associated with this result, and no conclusion can be drawn regarding the location origin of ST283 from the currently available dataset.

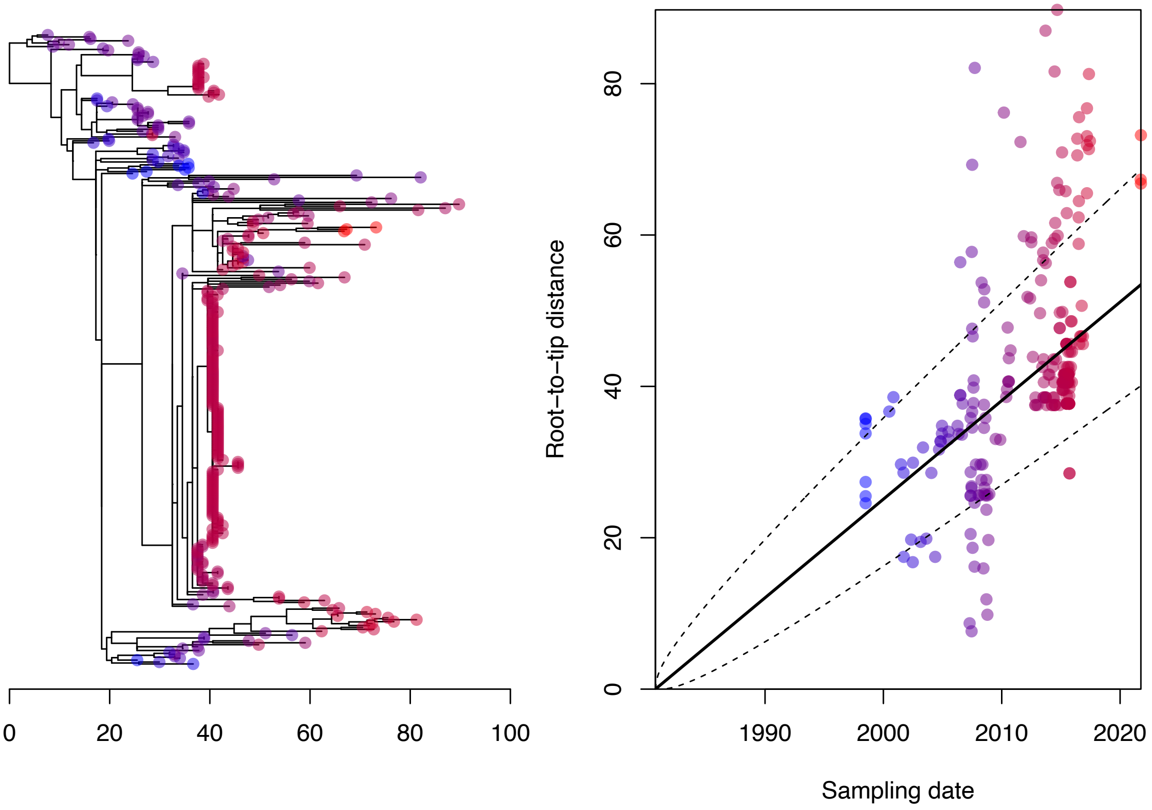

Fig A. Root-to-tip linear regression analysis based on the maximum likelihood (ML) phylogenetic tree (left), which demonstrates a relatively weak yet supported correlation (R^2 = 0.22, $p < 0.0001$) between date of sampling and genetic divergence (right).

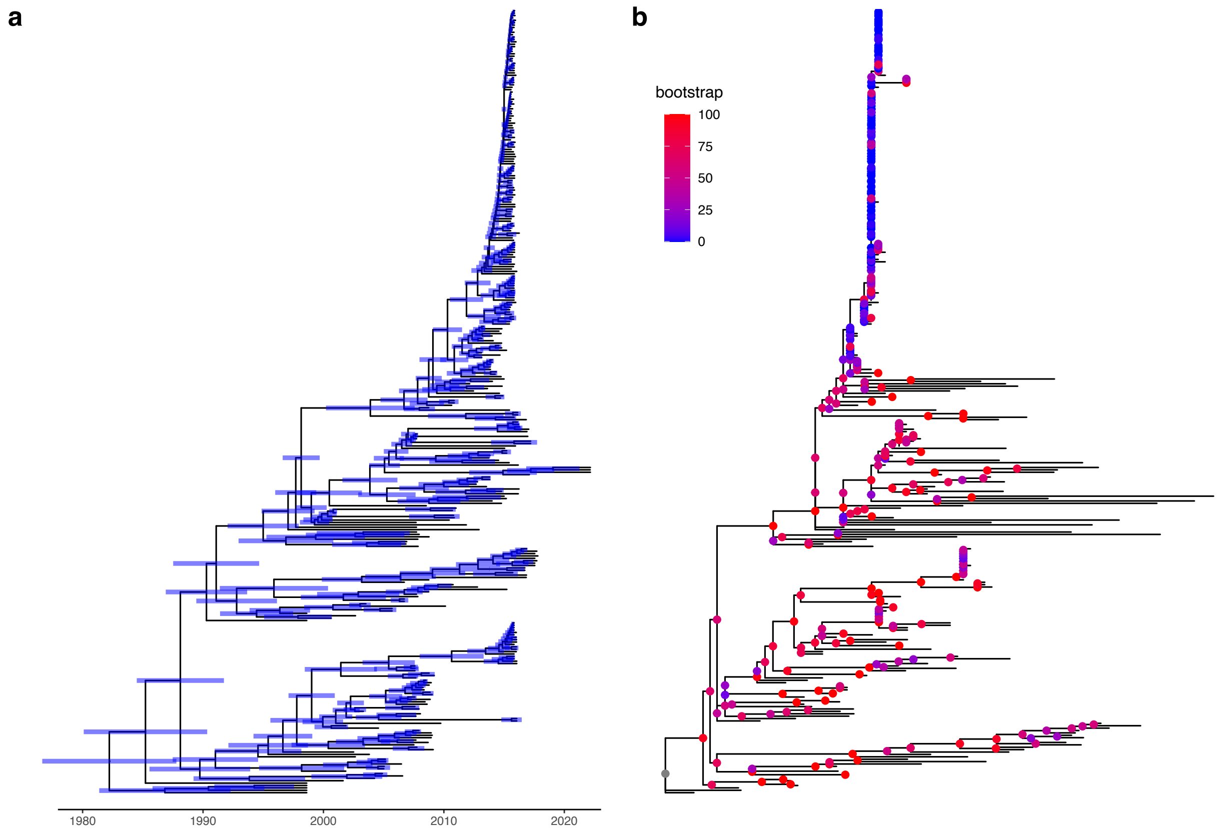

Fig B. Node age confidence intervals and bootstrap node support for GBS ST283. (a) Inferred node age 95% highest posterior density confidence intervals are displayed as blue bars in the time-scaled phylogenetic tree. **(b)** Unrooted maximum likelihood phylogenetic tree inferred from the 1214 SNP alignment (GTR gamma, 100 bootstrap replications using RAxML v8.2.12) showing bootstrap node support values.

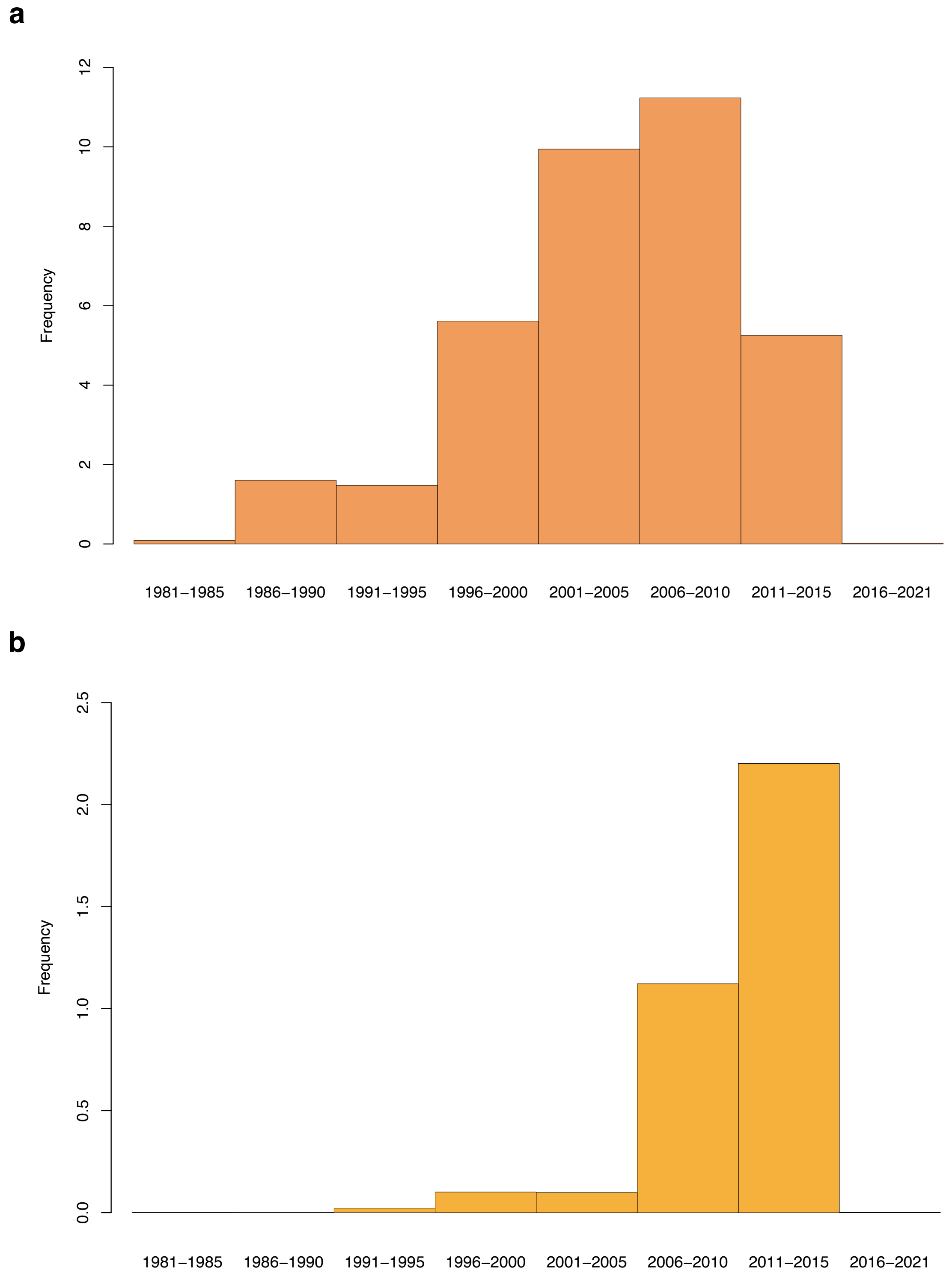

Fig C. International (a) and intercontinental (b) transitions. Frequency of transitions represent Markov jumps from the Bayesian stochastic search variable selection analysis. Intercontinental transitions **(b)** include only transitions to or from Asia and Brazil, the United Kingdom, or the United States.

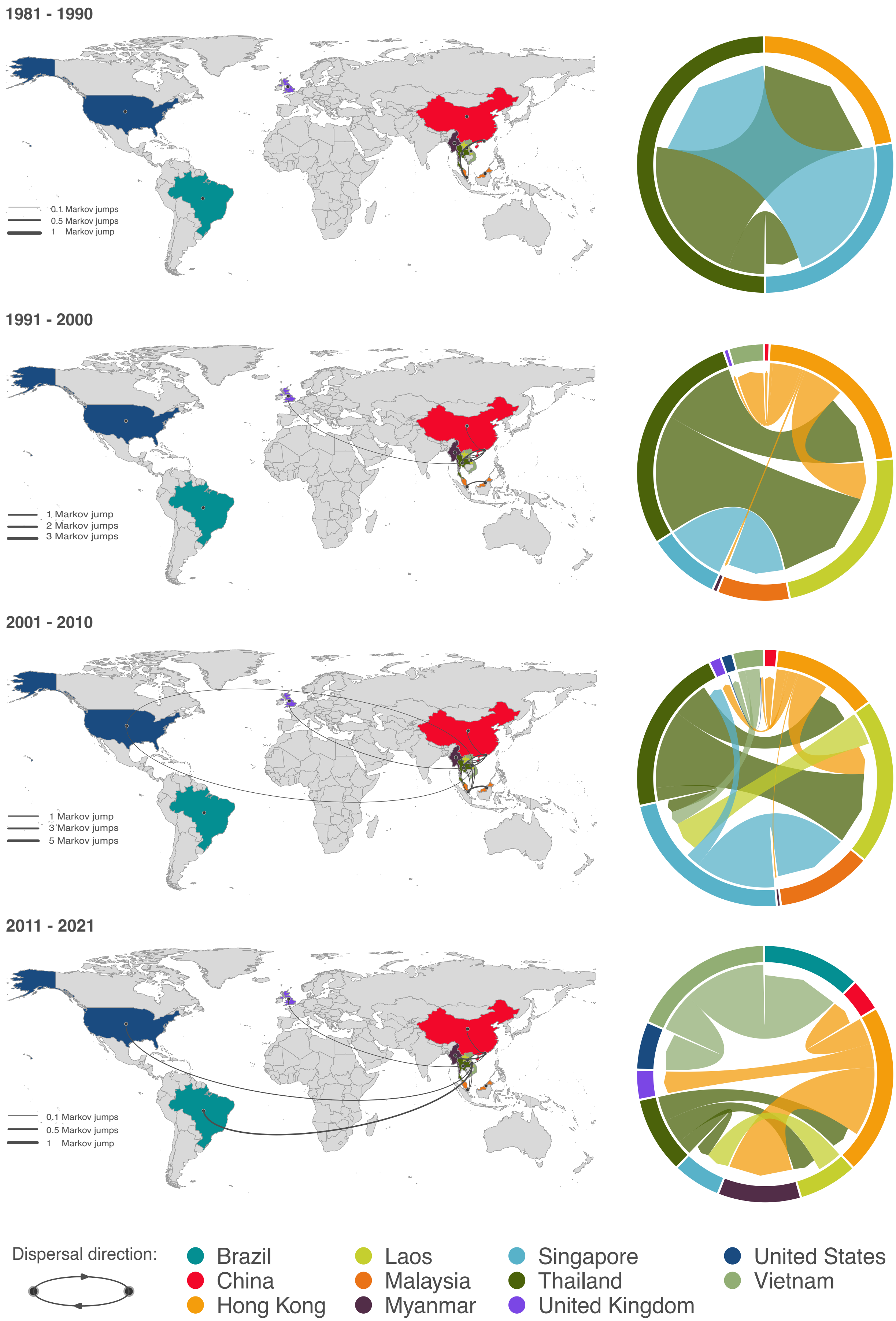
1981 - 1990
0.1 Markov jumps
0.5 Markov jumps
1 Markov jump
1991 - 2000
1 Markov jump
2 Markov jumps
3 Markov jumps
2001 - 2010
1 Markov jump
3 Markov jumps
5 Markov jumps
2011 - 2021
0.1 Markov jumps
0.5 Markov jumps
1 Markov jump
Dispersal direction:
Brazil
China
Hong Kong
Laos
Malaysia
Myanmar
Singapore
Thailand
United Kingdom
United States
Vietnam

Fig D. Discrete phylogeographic analysis of the dispersal history of GBS ST283 between 1981 and 2021. Intracontinental and intercontinental transition events are inferred as Markov jumps. Maps display transition events by decade and are accompanied by circular migration flow plots, in which transitions out of a country are represented by arrows originating at the outer ring and ending in an arrowhead offset from the destination country. Arrow width is proportional to the magnitude of the Markov jumps. Only transition events associated with standard (non-adjusted) Bayes factor support > 20 are displayed, a threshold value corresponding to strong statistical support (see the Methods section for further detail).

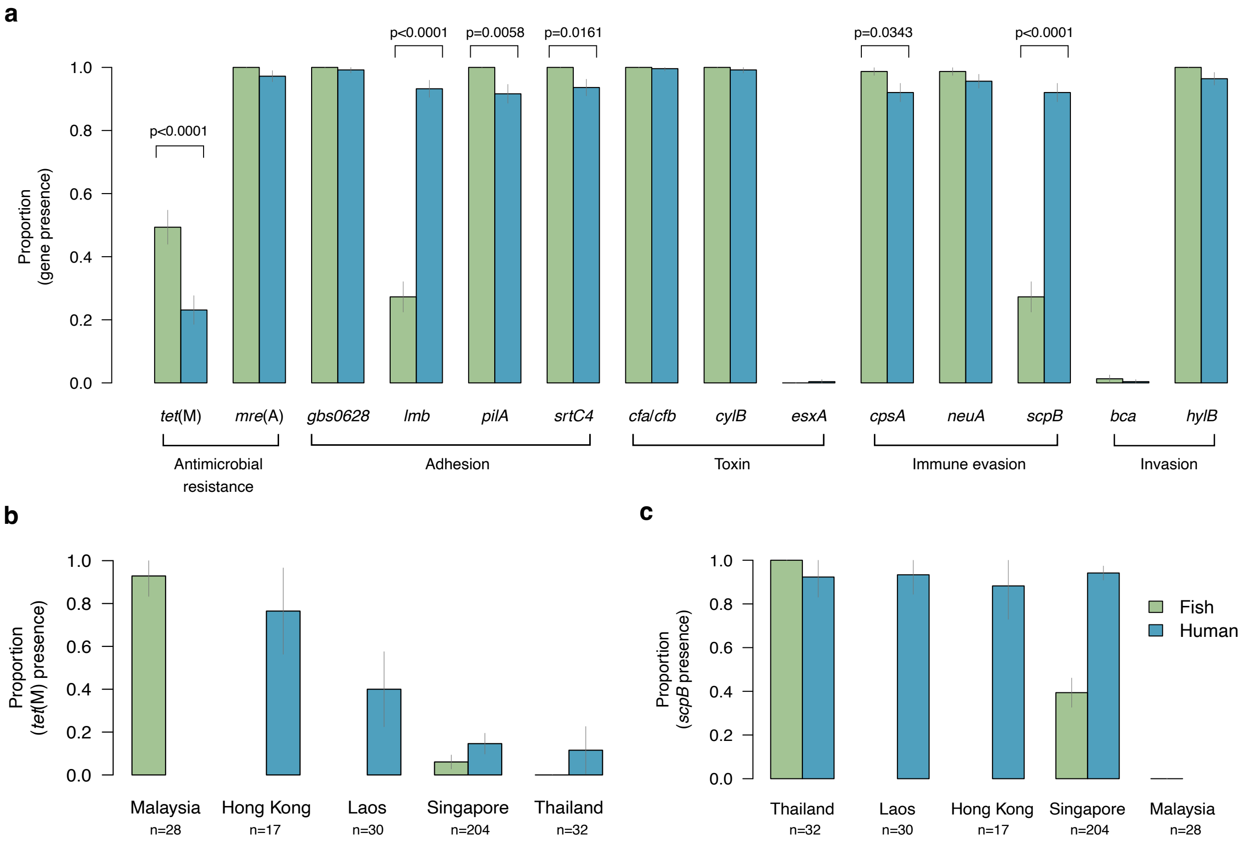

Fig E. Variable patterns of antimicrobial resistance and virulence factor gene presence in GBS ST283 from humans and fish. Empirical correlations between carriage of antimicrobial resistance and virulence factor genes and both host and sampling country for the isolates were evaluated by Fisher's exact test. **(a)** Carriage of antimicrobial resistance and virulence factor genes in isolates (n = 328) originating from fish and humans (Fisher's exact test; p-values lower than the threshold for significance (<.05) are shown). Gray bars represent 95% proportion confidence intervals. **(b,c)** Proportion of isolates in each country carrying the **(b)** *tet*(M) tetracycline resistance and **(c)** *scpB* virulence factor genes. Countries in which 10 or more isolates were tested are displayed. Host is not represented when no isolates from that host were sampled in that country. Gray bars represent 95% proportion confidence intervals. Fisher's exact test with Bonferroni correction for country pairs are **(b)** *tet*(M): Malaysia-Laos p = 0.0012, Malaysia-Singapore p < 0.0001, Malaysia-Thailand p<0.0001, Hong Kong-Singapore p < 0.0001, Hong Kong-Thailand p = 0.0002, Laos-Singapore p = 0.0483; and **(c)** *scpB*: Malaysia-Hong Kong p < 0.0001, Malaysia-Laos p < 0.0001, Malaysia-Singapore p < 0.0001, Malaysia-Thailand p < 0.0001. All other country associations are not significant.

References in S1 Text

1. Andrews, S. FastQC: A Quality Control Tool for High Throughput Sequence Data [Online]. 2010; Available at: http://www.bioinformatics.babraham.ac.uk/projects/fastqc/.

2. Prjibelski A, Antipov D, Meleshko D, Lapidus A, Korobeynikov A. Using SPAdes De Novo Assembler. Curr Protoc Bioinforma **2020**; 70. Available at: https://onlinelibrary.wiley.com/doi/10.1002/cpbi.102. Accessed 29 May 2022.

3. Mehershahi KS, Hsu LY, Koh TH, Chen SL. Complete Genome Sequence of Streptococcus agalactiae Serotype III, Multilocus Sequence Type 283 Strain SG-M1. Genome Announc **2015**; 3:e01188-15.

4. Harris SR. SKA: Split Kmer Analysis Toolkit for Bacterial Genomic Epidemiology. Genomics, 2018. Available at: http://biorxiv.org/lookup/doi/10.1101/453142. Accessed 29 May 2022.

5. Croucher NJ, Page AJ, Connor TR, et al. Rapid phylogenetic analysis of large samples of recombinant bacterial whole genome sequences using Gubbins. Nucleic Acids Res **2015**; 43:e15–e15.

6. Page AJ, Taylor B, Delaney AJ, et al. SNP-sites: rapid efficient extraction of SNPs from multi-FASTA alignments. Microb Genomics **2016**; 2. Available at: https://www.microbiologyresearch.org/content/journal/mgen/10.1099/mgen.0.000056. Accessed 29 May 2022.

7. Bland C, Ramsey TL, Sabree F, et al. CRISPR Recognition Tool (CRT): a tool for automatic detection of clustered regularly interspaced palindromic repeats. BMC Bioinformatics **2007**; 8:209.

8. Biswas A, Staals RHJ, Morales SE, Fineran PC, Brown CM. CRISPRDetect: A flexible algorithm to define CRISPR arrays. BMC Genomics **2016**; 17:356.

9. Edgar RC. PILER-CR: Fast and accurate identification of CRISPR repeats. BMC Bioinformatics **2007**; 8:18.

10. Puterová J, Martínek T. digIS: towards detecting distant and putative novel insertion sequence elements in prokaryotic genomes. BMC Bioinformatics **2021**; 22:258.

11. Seemann, T. Abricate. Available at: https://github.com/tseemann/abricate.

12. Feldgarden M, Brover V, Gonzalez-Escalona N, et al. AMRFinderPlus and the Reference Gene Catalog facilitate examination of the genomic links among antimicrobial resistance, stress response, and virulence. Sci Rep **2021**; 11:12728.

13. Stamatakis A. RAxML version 8: a tool for phylogenetic analysis and post-analysis of large phylogenies. Bioinformatics **2014**; 30:1312–1313.

14. Didelot X, Croucher NJ, Bentley SD, Harris SR, Wilson DJ. Bayesian inference of ancestral dates on bacterial phylogenetic trees. Nucleic Acids Res **2018**; 46:e134–e134.

15. Yu G, Smith DK, Zhu H, Guan Y, Lam TT. GGTREE : an R package for visualization and annotation of phylogenetic trees with their covariates and other associated data. Methods Ecol Evol **2017**; 8:28–36.

16. Lemey P, Rambaut A, Drummond AJ, Suchard MA. Bayesian phylogeography finds its roots. PLoS Comput Biol **2009**; 5:e1000520.

17. Suchard MA, Lemey P, Baele G, Ayres DL, Drummond AJ, Rambaut A. Bayesian phylogenetic and phylodynamic data integration using BEAST 1.10. Virus Evol **2018**; 4:vey016.

18. Rambaut A, Drummond AJ, Xie D, Baele G, Suchard MA. Posterior Summarization in Bayesian Phylogenetics Using Tracer 1.7. Syst Biol **2018**; 67:901–904.

19. Vrancken B, Mehta SR, Ávila-Ríos S, et al. Dynamics and Dispersal of Local Human Immunodeficiency Virus Epidemics Within San Diego and Across the San Diego-Tijuana Border. Clin Infect Dis Off Publ Infect Dis Soc Am **2021**; 73:e2018–e2025.

20. Trovão NS, Baele G, Vrancken B, et al. Host ecology determines the dispersal patterns of a plant virus. Virus Evol **2015**; 1:vev016.

21. Kass R, Raftery A. Bayes factors. J Am Stat Assoc **1995**; 90:773–795.

22. Dellicour S, Baele G, Dudas G, et al. Phylodynamic assessment of intervention strategies for the West African Ebola virus outbreak. Nat Commun **2018**; 9:2222.

23. Zhang Z, Nishimura A, Trovão NS, et al. Accelerating Bayesian inference of dependency between complex biological traits. **2022**; Available at: https://arxiv.org/abs/2201.07291. Accessed 31 October 2022.

24. Zhang Z, Nishimura A, Bastide P, et al. Large-scale inference of correlation among mixed-type biological traits with phylogenetic multivariate probit models. Ann Appl Stat **2021**; 15.

Available at: https://projecteuclid.org/journals/annals-of-applied-statistics/volume-15/issue-1/Large-scale-inference-of-correlation-among-mixed-type-biological-traits/10.1214/20-AOAS1394.full. Accessed 31 October 2022.
