# Supplementary material for: Dispersal history and bidirectional human-fish host switching of invasive, hypervirulent Streptococcus agalactiae sequence type 283
Source: PLOS Glob Public Health. 2023 Oct 19;3(10):e0002454. doi: 10.1371/journal.pgph.0002454 (PMC10586614; doi:10.1371/journal.pgph.0002454)
Supplement: Attachment — Submitted filename: Schar_D_et_al_PGPH-D-23-01077_Response to Reviewers.docx [file pgph.0002454.s003.docx]

Dear Editors,

We appreciate the opportunity to submit a revised manuscript. Please find below a detailed point-by-point response to the editors’ and reviewers’ comments. Editors’ and reviewers’ comments are in black, and our response is in blue. Line number references correspond to the revised track change manuscript.

**Editor Comments**

Potential Copyright Issues:

Fig 1&2: please (a) provide a direct link to the base layer of the map (i.e., the country or region border shape) and ensure this is also included in the figure legend; and (b) provide a link to the terms of use / license information for the base layer image or shapefile. We cannot publish proprietary or copyrighted maps (e.g. Google Maps, Mapquest) and the terms of use for your map base layer must be compatible with our CC-BY 4.0 license.

Note: if you created the map in a software program like R or ArcGIS, please locate and indicate the source of the basemap shapefile onto which data has been plotted.

If your map was obtained from a copyrighted source please amend the figure so that the base map used is from an openly available source. Alternatively, please provide explicit written permission from the copyright holder granting you the right to publish the material under our CC-BY 4.0 license.

Please note that the following CC BY licenses are compatible with PLOS license: CC BY 4.0, CC BY 2.0 and CC BY 3.0, meanwhile such licenses as CC BY-ND 3.0 and others are not compatible due to additional restrictions.

If you are unsure whether you can use a map or not, please do reach out and we will be able to help you. The following websites are good examples of where you can source open access or public domain maps: 
* U.S. Geological Survey (USGS) - All maps are in the public domain. (<http://www.usgs.gov>) 
* PlaniGlobe - All maps are published under a Creative Commons license so please cite “PlaniGlobe, <http://www.planiglobe.com>, CC BY 2.0” in the image credit after the caption. (<http://www.planiglobe.com/?lang=enl>) 
* Natural Earth - All maps are public domain. (<http://www.naturalearthdata.com/about/terms-of-use/>)

All base layer maps throughout the manuscript originate from the Natural Earth website and are in the public domain (<https://www.naturalearthdata.com/about/terms-of-use/>). The direct link to this base layer is: https://www.naturalearthdata.com/downloads/10m-cultural-vectors/10m-admin-0-countries/

Your manuscript has now been assessed by two reviewers and their feedback can be found below. Please consider the reviewers comments and revise your manuscript, particularly comments suggesting that you include a section on the quality and characteristics of the genomes used in the study.

Thank you. We have added a new section to provide additional details regarding the quality and characteristics of the genomes analyzed in our study. The full characteristics of all sequences analyzed in this study are provided in Supplementary Dataset S1.

**Reviewer Comments**

Reviewer #1: The authors have collated genomic information from a human and fish pathogenic clone of Streptococcus agalactiae ST283 to study its geographic spread and host dynamics. They have illustrated that the clone experienced increased dispersal to regions around the world since its emergence in the 1980’s. They also highlight that isolates have likely transmitted from humans to fish more frequently than from fish to humans. The data is somewhat biased since it is missing data from certain regions or contains more isolates from specific hosts or countries, however, this has been well documented in the text and statistical analyses have been carried out to account for and/or prove the effect of these biases. These findings highlight the potential for movement of virulent bacterial strains between aquaculture animals and susceptible human populations resulting in fish mortality and human infections. The authors advocate for further genomic surveillance and typing of Streptococcus agalactiae to further this work and help find mechanisms for reducing the spread of this pathogen. The manuscript is well written and most supplementary material and data has been included.

**Response to Reviewer #1**

We thank the reviewer for the thoughtful and detailed review.

I have some comments and questions for the authors to consider.

Most important are:
1. I noticed that a lot of the genomes with variable AMR and virulence gene content in Figure 1 compared to the rest of the dataset were from one country, Laos. Are these isolates all from the same study and is it possible that the assemblies from these data were not as good resulting in missing data?

R1.1 Thank you. Our data derives almost exclusively from public repositories (n=325/328; 99.1%) and were obtained primarily as assemblies (n=317/328; 96.7%). Isolates from both Thailand and Laos exhibited greater variability in gene presence/absence compared with isolates sampled in other countries. Because we obtained the majority of sequences—including all of the sequences from Thailand and Laos—as assemblies, we do not have sequence read quality and assembly statistics for these sequences. We address this limitation in the Discussion section and comment—as the reviewer has suggested—on the potential for sequencing and assembly quality to impact our findings (L335-338).

“Third, whole genome sequences were primarily obtained as assemblies. The quality of genomes generated across differing sequencing and assembly methods could not be assessed. While genome quality could influence our findings, these genomes have been utilized in prior analyses.”

2. Can you include read quality and assembly statistics in the supplementary material?

R1.2 Thank you. We have included in Supplementary Dataset S1 (available at 10.5281/zenodo.8345450) the read quality and assembly statistics for the 11 sequences we obtained as sequence reads (fastq) and independently assembled.

Minor comments:
Line 58: “reduce impact from” -> “reduce the impact of”

R1.3 Thank you. We have changed to “reduce the impact of” accordingly.

Line 125: Consider expanding HPD for non-specialists.

R1.4 Thank you. We have added “highest posterior density” on first use of this abbreviation in this line.

Line 167: Are some of these genes intrinsic to Streptococcus agalactiae as a whole?

R1.5 We are not aware that any of the antimicrobial resistance genes we identified are intrinsic to *Streptococcus agalactiae*. Notably, the functionality of the *mre*(A) gene in GBS is uncertain – a point which we have included in the Discussion section (L342-346):

“Recent work reported that despite nearly uniform carriage of *mre*(A) in a collection of ST283 isolates, none expressed resistance to macrolides [16]. In *Streptococcus agalactiae*, the *mre*(A) gene may serve a metabolic function, whereas when cloned into *E.coli*, *mre*(A) conferred macrolide resistance [28]. Additional study is needed to elucidate the phenotypic resistance profiles of ST283 and correlation with antimicrobial resistance genes.”

Figure 1: The names of AMR genes and virulence genes could be made bigger.

R1.6 Thank you. Space constraints in this figure precluded increasing the font size for gene labels while maintaining the visual integrity of the entire figure. However, PLoS Global Public Health figure viewing panes allow for manual zoom that will magnify labels for ease of readability.

Figure S2: What does unrooted mean in this case?

R1.7 The maximum likelihood phylogenetic tree is inferred without reference to a common ancestor and is thus referred to as unrooted. We use an unrooted tree to show the phylogenetic relationship among genomes in the study through bootstrap node supports without implying an evolutionary direction over time inherent in rooted phylogenies.

Figure S4: I don't think this figure has been referenced in the text

R1.8 Thank you for calling our attention to this omission. We have added a reference to Supplementary Figure S4 in the main text (L410-416):

“In the discrete phylogeographic reconstruction, country transitions were estimated as Markov jump counts and reported along with both standard (Supplementary Figure S4) Bayes factor (BF) support values (ratio of posterior over prior odds and interpreted as a measure of the strength of evidence for the alternate hypothesis) and an adjusted (Figure 2) Bayes factor (BF_adj_) supports [45] accounting for sample size disparity.”

Reviewer #2: Dear authors, attached are some suggestions for your consideration.

**Response to Reviewer #2**

We greatly appreciate the suggestions from reviewer #2, which have improved the clarity with which we communicate our findings and enhanced the context for these findings.

Line 71

The authors are encouraged to consider the following references if they find it appropriate.

Barkham, T., Tang, W. Y., Wang, Y. C., Sithithaworn, P., Kopolrat, K. Y., & Worasith, C. (2023). Human Fecal Carriage of Streptococcus agalactiae Sequence Type 283, Thailand. Emerging Infectious Diseases, 29(8), 1627.

Zhou, Y., Zhou, S., Peng, J., Min, L., Chen, Q., & Ke, J. (2023). Bacterial distribution and drug resistance in blood samples of children in Jiangxi Region, 2017–2021. Frontiers in Cellular and Infection Microbiology, 13, 1163312.

Sirimanapong, W., Phước, N. N., Crestani, C., Chen, S., & Zadoks, R. N. (2023). Geographical, Temporal and Host-Species Distribution of Potentially Human-Pathogenic Group B Streptococcus in Aquaculture Species in Southeast Asia. Pathogens, 12(4), 525.

Luangraj, M., Hiestand, J., Rasphone, O., Chen, S. L., Davong, V., Barkham, T., ... & Keoluangkhot, V. (2022). Invasive Streptococcus agalactiae ST283 infection after fish consumption in two sisters, Lao PDR. Wellcome open research, 7.

Wang, J., Zhang, Y., Lin, M., Bao, J., Wang, G., Dong, R., ... & Pan, X. (2023). Maternal colonization with group B Streptococcus and antibiotic resistance in China: systematic review and meta-analyses. Annals of clinical microbiology and antimicrobials, 22(1), 5.

R2.1 We thank the reviewer for calling our attention to these recently published references, and have added them throughout the manuscript as follows:

- Barkham, T. et al. (L255)
- Sirimanapong, W. et al. (L98 and L302 and L322)
- Luangraj, M. et al. (L103)
- Wang, J. et al. (L346-348)

Line 153

It is possible to consider incorporating the availability and impact of the effort and the ability to obtain human or fish sequences in these results?

R2.2 Our dataset comprises all publicly available ST283 sequences to date obtained through searches of both genomic repositories and the literature as well as additional sequences curated through research networks. Despite this, the resulting dataset is indeed characterized by sampling heterogeneity, including in the host sampled (human and fish). In our study, we account for this disparity in host sampling (more human than fish isolates) by working on downsampled phylogenies with equal numbers of isolates originating from human and fish hosts and further conducting three additional analyses to assess potential impact from sampling date and phylogenetic diversity associated with host type. This is articulated in the Methods section (L418-426), the Figure 3 legend, and in the Results section (L155-161):

“Heterogeneous host sampling distribution in the dataset precluded inference of supported Markov jumps between hosts… To account for this sampling bias, ancestral host state transitions were inferred by maximum likelihood estimation on downsampled phylogenies”

Line 215 Figure 1 and “Supplementary Figure S2a”.

The suggestion is to clarify and standardize the names of supplementary information and supplementary figures. It is not easy to track the information as it is currently named.
The suggestion is to systematically list and number the information so that the reader can quickly identify it.

R2.3 We follow the PLoS Global Public Health guidelines (https://journals.plos.org/globalpublichealth/s/supporting-information). Specifically, “supplementary information” refers to the supplementary information document that includes Supplementary Methods, Supplementary Tables S1 and S2, Supplementary Figures S1 to S5, and Supplementary References. These are referred to systematically throughout the main body of the manuscript. In the example provided, we use “Supplementary Figure S2a” (L126) to point the reader specifically to panel “a” of Supplementary Figure S2. We are pleased to remove any reference to figure panels in the manuscript according to editorial direction.

Line 341

It is suggested to include more detailed information about the genomes, sequencing methods, sequence quality parameters, and criteria for inclusion and exclusion of genomes in the analysis. Certain aspects, such as the reference sequence and whether the available genomes are fully assembled or assembled from published partial sequences, sequencing methods should be included in the Materials and Methods section. It is suggested that certain elements of the materials and methods, as described in 'Click here to access/download Supporting Information: Schar_D_et_al_PLOSGPH_Supplementary_Information.pdf', should be included in the document to achieve a comprehensive understanding of it.

R2.4 We thank the reviewer for this helpful suggestion. We have added a new Methods sub-section entitled “Whole genome sequence analysis” to provide additional details regarding the quality and characteristics of the genomes in our study. We have also added details from the Supplementary Methods into the main body of the manuscript Methods section, as suggested by the reviewer.

We have further added sequence quality parameters and assembly statistics (Phred score; number of contigs; largest contig; GC%; N50; L50) in Supplementary Dataset S1 (available at 10.5281/zenodo.8345450). As noted in R1.1, sequences were obtained primarily as assemblies (n=317/328; 96.7%), for which we are unable to assess sequence read quality and assembly statistics. We note in the Methods section that all ST283 genomes with corresponding sampling date and location were included in the study, and we have now added an explicit reference to exclusion of genomes without these corresponding data (L367-369). Finally, we specify that assemblies were mapped to the reference sequence SG-M1 (accession CP012419.2) (L392).

Line 359

Figure 1: The suggestion is that the table displaying the presence or absence of antimicrobial resistance genes (blue) and virulence factor genes (red) for each isolate in the study (Table "D") be considered. The suggestion is to condense the information in the figure/table. Specifically, for the table of resistance genes, all sequences with the same pattern can be removed, retaining only a few representative ones. he letters are too small.

R2.5 We appreciate the reviewer’s suggestion to remove sequences and gene presence/absence data in Figure 1, panel c. However, visual representation of the full dataset enables readers to (1) identify patterns in the genomic data that aid in interpretation of the findings presented; (2) quickly ascertain the geographic distribution of sequences in the dataset; and (3) appreciate the geographic sampling heterogeneity inherent to the dataset. Finally, removing sequences with similar patterns could be perceived as distortion of the underlying dataset.

The suggestion is to thoroughly discuss the effects of sample availability on the statement regarding the transmission of bacteria from humans to fish or from fish to humans. Additionally, it is recommended to provide a basis for the loss of virulence and resistance genes as an evolutionary strategy.

R2.6 We thank the reviewer for these suggestions. We discuss sampling heterogeneity inherent to the dataset in the context of our findings regarding host switching (L241-247):

“We observe more frequent human-to-fish than fish-to-human host switching – a trend that remains consistent when restricting the analysis to include the same number of sampled hosts in each year and when controlling for differences in phylogenetic diversity associated with each host type. Heterogeneous effort inherent to this dataset in sampling practices (point prevalence, event-based, and opportunistic) could affect these results. As such, these findings reflect the data currently available, and we cannot ascertain to what extent a different sampling effort would yield different results, which calls for further investigation.”

We further comment on the potential effects of sampling heterogeneity on our findings (L332-335):

“Second, our dataset is characterized by sampling heterogeneity. While we account for this heterogeneous sampling effort in our analyses, the disparity in host, location, and sampling date could influence our findings.”

Finally, we add a discussion of evolutionary strategies as a basis for the loss of virulence and resistance genes (L298-304):

“Antimicrobial resistance and virulence factor genes were lost more frequently than gained throughout the evolutionary history of ST283. An active rate of gene change in ST283 is consistent with GBS as a pathogen under evolutionary pressure, with recombination and mobile elements contributing to a shifting genome composition generating new, niche-adapted lineages [11,23,26]. Gene loss may facilitate rapid adaptation to the functional requirements of host suitability, for example, reducing the metabolic burden of gene expression, enhancing fitness and conferring a selective advantage in such niche-adapted lineages [23].”
